# Moderate warming promotes growth and flavonoid biosynthesis in the altitudinal medicinal plant *Gentiana lawrencei* var. *farreri*

**DOI:** 10.3389/fpls.2026.1806606

**Published:** 2026-04-24

**Authors:** Guopeng Chen, Jie Zhang, Xianmei Yin, Xuemei Wu, Lame Zeren, Zhuoma Deqing, Chenghui Wang, Yue Xu, Jiemin Wang, Hongmei Jia, Rong Ding, Rui Gu

**Affiliations:** 1 School of Ethnic Medicine, Chengdu University of Traditional Chinese Medicine, Chengdu, China; 2 State Key Laboratory of Southwestern Chinese Medicine Resources, Chengdu University of Traditional Chinese Medicine, Chengdu, China; 3 Chongqing Traditional Chinese Medicine Hospital, Chongqing, China

**Keywords:** antioxidant capacity, endangered plants, flavonoids, soluble proteins, soluble sugars, temperature

## Abstract

High-altitude endangered medicinal plants face increasing extinction risk, and *ex situ* conservation through low-altitude cultivation represents a key protective strategy. Low temperature is a defining feature of altitudinal habitats. However how moderate warming influences growth performance and secondary metabolite accumulation in cold-adapted medicinal plants remains poorly understood. We investigated the altitudinal medicinal plant *Gentiana lawrencei* var. *farreri* (*G. farreri*) under different day/night temperature regimes, integrating morphological, physiological, transcriptomic and metabolomic analyses. Moderate warming (25/15 °C) significantly increased leaf area, root length, and shoot and root dry biomass, while reducing soluble sugar and protein contents. Antioxidant enzyme activities, as well as proline and malondialdehyde levels, were decreased. Weighted gene co-expression network analysis identified a temperature-responsive module closely associated with multiple stress-related traits, in which the plant–pathogen interaction pathway was prominently enriched. Key regulatory nodes, including FLS2 and CDPK, showed temperature-dependent expression patterns. Levels of flavonoids, including *p*-coumaric acid, dihydromyricetin, delphinidin and cyanidin, were markedly increased under moderate warming. These results demonstrate that moderate warming alleviates temperature limitation on growth and promotes flavonoid accumulation in *G*. *farreri*. Our findings provide insight into the physiological and molecular acclimation of alpine medicinal plants to moderate warming and offer a scientific basis for optimizing low-altitude *ex situ* cultivation under climate warming.

## 1 Introduction

High-altitude ecosystems harbour unique reservoirs of plant biodiversity, including numerous medicinal species with distinctive pharmacological properties. Altitudinal medicinal plants have long been used in traditional medicine and are increasingly recognised as valuable resources for modern drug discovery. However, these species are particularly vulnerable to climate change. Rising temperature, one of the most pronounced environmental shifts in high-altitude regions, has intensified in recent decades with the increasing frequency of extreme climatic events (Sloat et al., 2020; Harrington et al., 2021). Climate warming alters plant growth, survival, and the biosynthesis of bioactive compounds, posing significant challenges for the conservation and sustainable utilisation of altitudinal medicinal resources (Yang et al., 2011; Karimi et al., 2020; Wen et al., 2022).

*Gentiana lawrencei* var. *farreri* (*G. farreri*) is a cold-adapted, endangered altitudinal medicinal plant widely distributed in high-mountain meadows, shrublands and riparian habitats at elevations of 2,410–4,600 m. It is a perennial herb that flowers and fruits from August to October (Fu et al., 2018). G. *farreri* serves as a principal raw material for the traditional Tibetan medicine formulation Shi Wei Long Dan Hua Ke Li. The leaves and flowers are used medicinally and have been widely applied in Tibetan medicine for the treatment of pneumonia, hepatitis, and both acute and chronic bronchitis (Wei et al., 2023). These environments are characterised by low effective accumulated temperature and a mean annual temperature of approximately 4 °C (Fu et al., 2016).

*Ex situ* conservation through low-altitude cultivation has been proposed as a practical strategy to mitigate extinction risk; however, a reduction in altitude inevitably exposes plants to moderate warming. Despite its ecological and medicinal importance, how temperature elevation modulates growth, stress resistance and the accumulation of key pharmacologically active compounds in *G. farreri* remains largely unresolved.

Temperature is a key environmental determinant of plant geographic distribution, photosynthetic performance and biomass accumulation. Photosynthetic rate (Pn) is typically constrained at both low and high temperatures, reaching an optimum at intermediate temperatures. Altitudinal plants often exhibit higher photosynthetic capacity than their lowland counterparts, reflecting adaptation to cold environments. In high-altitude regions, low temperature has been reported to increase Pn in herbaceous species by approximately 16% (Zhai et al., 2024). By contrast, in some species Pn increases with rising temperature, highlighting strong interspecific variation in thermal responses. Temperature-dependent regulation of photosynthesis is closely linked to Rubisco activity: in cotton, Rubisco becomes inactivated when leaf temperature exceeds 35 °C (Salvucci and Crafts-Brandner, 2004a), whereas in Antarctic hairgrass (*Deschampsia antarctica*), inactivation occurs at temperatures above 20 °C (Salvucci and Crafts-Brandner, 2004b). These contrasting thresholds indicate that modulation of Rubisco activity is a central component of photosynthetic temperature adaptation.

A meta-analysis of terrestrial plants showed that experimental warming increased biomass by 12.3% overall, with a substantially greater response in woody species (26.7%) than in herbaceous plants (5.2%) (Lin et al., 2010). However, warming experiments have yielded divergent outcomes, with biomass increases reported in some species (Sullivan et al., 2008; Pernicová et al., 2024), decreases in others (Klein et al., 2007), and no detectable effects in some species (Saleska et al., 2002). These contrasting responses underscore strong species- and ecosystem-specific sensitivity to temperature. Plants in high-latitude and high-altitude ecosystems are expected to respond more positively to warming than those at lower latitudes, owing to more rapid warming rates and stronger low-temperature constraints on growth in these regions (Parmesan, 2007).

Temperature strongly influences plant physiology and secondary metabolism. In alpine species such as *Pedicularis punctata* and *Plantago major*, decreasing altitude and increasing temperature are associated with significant reductions in leaf protein, soluble sugar, proline, and abscisic acid (ABA) contents, accompanied by an increase in indole-3-acetic acid (IAA) levels (Rahman et al., 2020). Flavonoid biosynthesis is regulated by multiple environmental factors, with temperature considered one of the most critical (Guo et al., 2020). Under heat stress, plants often accumulate flavonoids and phenolic compounds to mitigate damage (Gooding et al., 2001). Flavonoids are synthesized via the phenylpropanoid pathway, with key enzymes including 4-coumarate:CoA ligase (4CL), Cinnamate 4-hydroxylase (C4H), and Chalcone synthase (CHS) (Dong and Lin, 2021). Moderate warming can modulate reactive oxygen species (ROS) production, resulting in either increased or decreased flavonoid accumulation. For example, the B-box transcription factor MdCOL4 is induced by high temperature and directly represses *MdANS* and *MdUFGT*, reducing anthocyanin content in apple (Dong and Lin, 2021). Similarly, the SG7 R2R3-MYB transcription factor CmMYB012 is upregulated under prolonged heat, suppressing flavonoid biosynthesis in chrysanthemum via direct repression of *CmFNS* (Zhou et al., 2021). In the medicinal plant *Justicia adhatoda* L., individuals growing at higher altitudes exhibit increased accumulation of phenolic and flavonoid compounds (Jamwal et al., 2023). In *G. farreri*, flavonoids represent key bioactive constituents, yet how temperature regulates their accumulation remains unknown.

In this study, we focused on the altitudinal medicinal plant *G. farreri*. Plants were grown under distinct day/night temperature regimes, and we assessed morphological traits, root architecture, photosynthate accumulation, antioxidant enzyme activity, and performed integrated transcriptomic and metabolomic analyses. Our aim was to elucidate the physiological and metabolic responses of *G. farreri* to temperature, identify conditions that optimize growth and bioactive compound accumulation, and provide a scientific basis for the cultivation and conservation of this endangered altitudinal species under climate change.

## 2 Materials and methods

### 2.1 Plant material

Seeds of *G*. *farreri* were collected in October 2023 from Hongyuan County, Aba Tibetan and Qiang Autonomous Prefecture, Sichuan Province, China (32°50′42.20″ N, 102°35′26.21″ E; 3,497 m a.s.l.). The collection site is characterised by a mean annual temperature of approximately 3 °C, with recorded maximum and minimum temperatures of 25 °C and −24 °C, respectively.

### 2.2 Experimental design

The experiment was conducted in Wenjiang District, Chengdu, Sichuan Province, China (30°42′18.9″ N, 103°49′46.1″ E; 647 m a.s.l.), under controlled conditions in a plant growth chamber. Seeds were soaked in distilled water for 24 h, surface-sterilized with 2% (w/v) sodium hypochlorite for 10 min, and rinsed thoroughly with distilled water (five to six times). Uniform seeds were sown in plastic pots containing a substrate mixture of peat and vermiculite (4:1, v/v), with ten seeds per pot. Peat soil, characterized by its loose and porous structure and high organic matter content, provides a sustained nutrient supply for plant growth. Vermiculite enhances water retention and aeration while supplying trace elements. The soil contained 120.38 mg kg^−1^ available nitrogen, 5.23 mg kg^−1^ available phosphorus, 453.00 mg kg^−1^ available potassium, 0.43 mg kg^−1^ available zinc, and 0.42 mg kg^−1^ available manganese, with an organic matter content of 326.55 g kg^−1^.

The experiment followed a completely randomized design with three day/night temperature regimes: 15/5 °C (T15), 20/10 °C (T20), and 25/15 °C (T25). Plants were grown under a 12 h light/12 h dark photoperiod, a light intensity of 40,000 lux, and relative humidity maintained at 65%. From sowing to sampling, plants were continuously grown in growth chambers under their respective temperature treatments. Each treatment consisted of 48 pots, giving a total of 144 pots. After emergence, seedlings were thinned to two plants per pot, and all treatments received identical water management. Plants were irrigated once per week with Hoagland nutrient solution. Samples were collected after 120 days of growth for subsequent analyses.

### 2.3 Measurement of plant and root morphological traits

Ten seedlings were randomly selected from each treatment. Roots were carefully excavated, washed free of substrate, and gently blotted dry. Leaves and root systems were scanned to obtain digital images. Leaf area was quantified using ImageJ software, while total root length, mean root diameter, root surface area, root volume, and root tip number were determined using the WinRHIZO root analysis system. Shoots and roots were separated and heat-treated at 105 °C for 30 min to terminate metabolic activity, followed by drying at 60 °C to constant weight. Dry biomass of shoots and roots was then recorded.

### 2.4 Chlorophyll content determination

Leaf samples (0.1 g fresh weight) were collected from ten seedlings per treatment and extracted with 20 mL of 95% (v/v) ethanol in darkness for 24 h until complete tissue decolourisation, with intermittent agitation. Absorbance of the extracts was measured at 649 and 665 nm using a UV–visible spectrophotometer.

Chlorophyll concentrations were calculated using the following equations:

Chlorophyll a (μg g^−1^) = 13.95 × A_665_ − 6.88 × A_649_.
Chlorophyll b (μg g^−1^) = 24.96 × A_649_ − 7.32 × A_665_.

Total chlorophyll content was expressed on a fresh-weight basis (μg g^−1^ FW) and calculated as:

Chlorophyll content = (pigment concentration × extract volume)/leaf fresh weight.

### 2.5 Determination of soluble sugar, soluble protein and proline contents

Soluble sugar content in leaves was determined using the anthrone colorimetric method. Fresh leaf tissue (0.1 g) was extracted in 25 mL of deionized water at 95 °C for 30 min. An aliquot of the extract (50 μL) was mixed with 400 μL of anthrone reagent (2 mg mL^−1^ in concentrated sulfuric acid) and incubated at 95 °C for 5 min. Absorbance was measured at 625 nm using a microplate reader, and soluble sugar content was calculated as previously described (Tang et al., 2022). Soluble protein content was quantified using the Coomassie Brilliant Blue G-250 dye-binding assay according to Xu et al (Xu et al., 2022). Proline content was determined by the acidic ninhydrin colorimetric method following the protocol of Bates et al (Bates et al., 1973).

### 2.6 Malondialdehyde content determination

Leaf tissue (160 mg fresh weight) was homogenized in liquid nitrogen and extracted with 2 mL of 0.1% (w/v) trichloroacetic acid (TCA). The homogenate was centrifuged at 10,000 *g* for 15 min, and 1 mL of the supernatant was mixed with 1 mL of thiobarbituric acid (0.5%, w/v) prepared in 20% (w/v) TCA. The reaction mixture was incubated in a boiling water bath (95 °C) and immediately cooled in an ice bath to terminate the reaction. After centrifugation, absorbance was measured at 532 and 600 nm. MDA concentration was calculated using the difference in absorbance (ΔA = A_532_ − A_600_) and an extinction coefficient of 155 mM^−1^ cm^−1^ (Silveira et al., 2021).

### 2.7 Antioxidant enzyme activity assays

The activities of superoxide dismutase (SOD), peroxidase (POD), and catalase (CAT) were determined using commercial assay kits according to the manufacturers’ instructions. Each treatment included three biological replicates. Fresh leaf tissue (0.1 g) was used for enzyme extraction following the kit protocols. Enzyme activities were quantified by measuring absorbance changes with a microplate reader and calculated as described previously (Li et al., 2023).

### 2.8 Determination of bioactive compound contents

For each treatment, three plants were selected, and leaves were collected and oven-dried at 60 °C. The determination of bioactive compounds was performed with slight modifications based on the method described by Cuo et al (Cuo et al., 2020). Gentiopicrin (an iridoid glycoside) and isoorientin (a flavonoid) were used as reference compounds. The contents of iridoid glycosides (gentiopicrin, loganic acid, swertiamarin, and sweroside) and flavonoids (isoorientin, isoscoparin-2″-β-D-glucopyranoside, and Isoscoparin) were quantified using the relative correction factor method.

### 2.9 RNA extraction and transcriptomic analysis

For each treatment, three independent plants were selected, and leaf samples were collected, immediately frozen in liquid nitrogen, and used for RNA extraction. Total RNA was isolated using the TRIzol reagent following a modified protocol described by Baute et al. (Baute et al., 2015). RNA quality and integrity were assessed prior to library construction. cDNA libraries were prepared and sequenced on the Illumina HiSeq platform to generate raw reads.

Raw sequencing data were quality-checked using FastQC (v0.11.4) and processed with Trimmomatic (v0.33) to remove low-quality reads and adaptor sequences. Due to the lack of a reference genome for *G*. *farreri*, high-quality clean reads were assembled *de novo* using Trinity (V2.11.0; https://github.com/trinityrnaseq/trinityrnaseq/wiki) (Grabherr et al., 2011).

Gene expression levels were quantified using fragments per kilobase of transcript per million mapped reads (FPKM), and differential expression analysis was performed with Cuffdiff. Differentially expressed genes (DEGs) were identified based on statistical significance thresholds.

Gene Ontology (GO) enrichment analysis and Kyoto Encyclopedia of Genes and Genomes (KEGG) pathway enrichment analysis were conducted using GOATOOLS and KOBAS, respectively. Weighted gene co-expression network analysis (WGCNA) was performed in R to construct co-expression networks of DEGs under temperature treatments and to identify gene modules associated with physiological traits.

### 2.10 Metabolomic analysis

Six plants per treatment were randomly selected for metabolomic profiling, and leaf samples were collected. Freeze-dried tissue (100 mg) was extracted with 500 μL of pre-cooled methanol–acetonitrile–water (2:2:1, v/v/v) containing 5 ppm 2-chlorophenylalanine as an internal standard, followed by centrifugation and filtration (0.22 μm).

Metabolites were separated using an ACQUITY UPLC HSS T3 column and analysed on a Thermo Orbitrap Exploris 120 mass spectrometer operated in both positive and negative ion modes. Data were processed with Compound Discoverer™ 3.3, and metabolites were identified by comparison with in-house and public databases, with a precursor mass tolerance of 15 ppm. Metabolites with a fold change ≥ 2 or ≤ 0.5 were considered significantly differential metabolites (SDMs).

### 2.11 Data analysis

Data were processed in Excel 2010. One-way ANOVA and graphical presentation were performed using GraphPad Prism 8. Differences among treatments were assessed using Tukey’s honestly significant difference (HSD) test. Differences were considered statistically significant at *p* < 0.05, and distinct letters indicate significant differences between treatments.

## 3 Results

### 3.1 Moderate warming promotes plant growth

**Figure 1:**
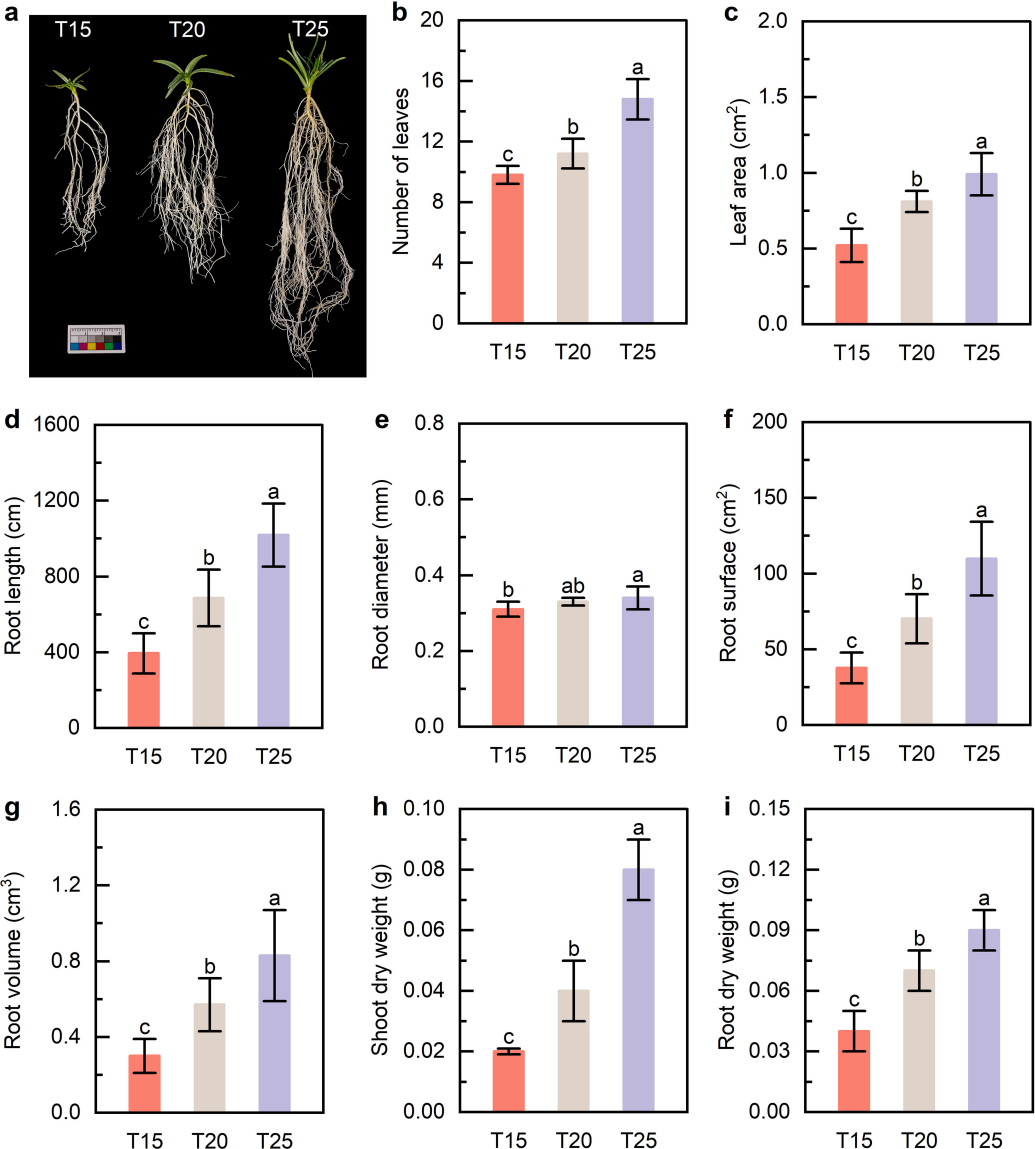
Morphological characteristics of *G*. *farreri* **(a)** Photograph of *G*. *farreri* plants. **(b, c)** Leaf number and leaf area. **(d–g)** Root length, root diameter, root surface area, and root volume. **(h)** Shoot dry weight. **(i)** Root dry weight. T15, T20, and T25 represent plants grown under different day/night temperature regimes of 15/5 °C, 20/10 °C, and 25/15 °C, respectively. Different lowercase letters indicate significant differences among treatments (*p* < 0.05).

Plant growth performance under different temperature treatments was systematically evaluated. Moderate warming increased leaf number, leaf area, and root length. Under T25, leaf number, leaf area, root length, and root diameter were significantly higher than under T20 and T15 (**Figures 1b–e**), although root diameter at T25 did not differ from T20. Root surface area, root volume, and shoot and root dry biomass were also highest at T25 (**Figures 1f–i**). These results indicate that moderate temperature elevation promotes both above- and belowground growth and enhances biomass accumulation.

### 3.2 Leaf chlorophyll, soluble sugar, and soluble protein

**Figure 2:**
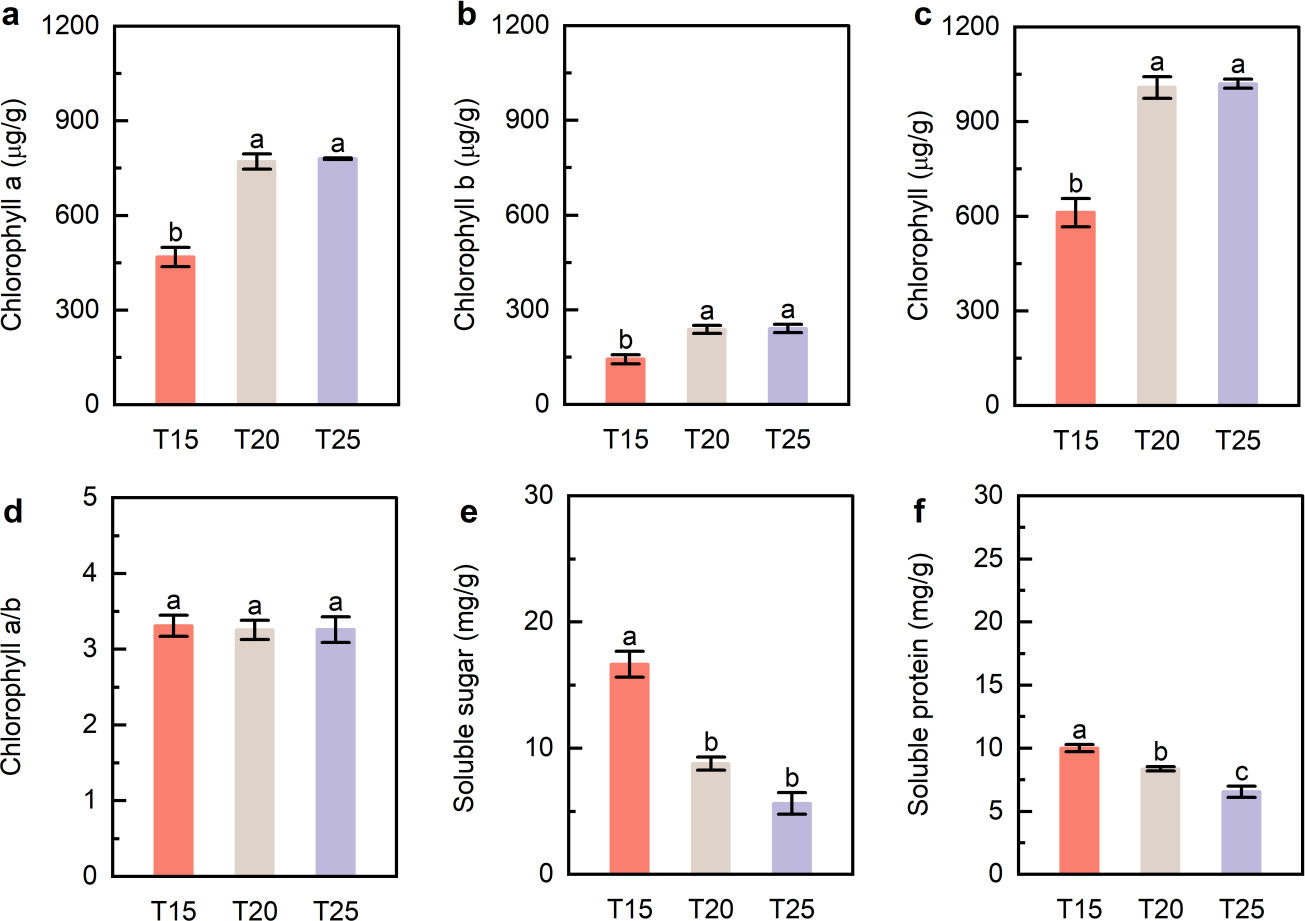
Leaf chlorophyll, soluble sugar, and soluble protein contents. **(a)** Chlorophyll a content. **(b)** Chlorophyll b content. **(c)** Total chlorophyll content. **(d)** Chlorophyll a/b ratio. **(e, f)** Soluble sugar and soluble protein contents. T15, T20, and T25 represent plants grown under different day/night temperature regimes of 15/5 °C, 20/10 °C, and 25/15 °C, respectively. Different lowercase letters indicate significant differences among treatments (*p* < 0.05).

Changes in leaf chlorophyll content and osmotic regulatory substances were evaluated under different temperature treatments. Chlorophyll a, chlorophyll b, and total chlorophyll were higher at T25 than at T15, but did not differ significantly from T20 (**Figures 2a–c**). The chlorophyll a/b ratio was similar across treatments (**Figure 2d**). Soluble sugar and protein contents were highest at T15 and decreased with increasing temperature (**Figures 2e, f**). Moderate warming (20–25 °C) enhances chlorophyll accumulation while reducing soluble sugar and protein levels.

### 3.3 Moderate warming reduces osmolyte accumulation and antioxidant enzyme activity

**Figure 3:**
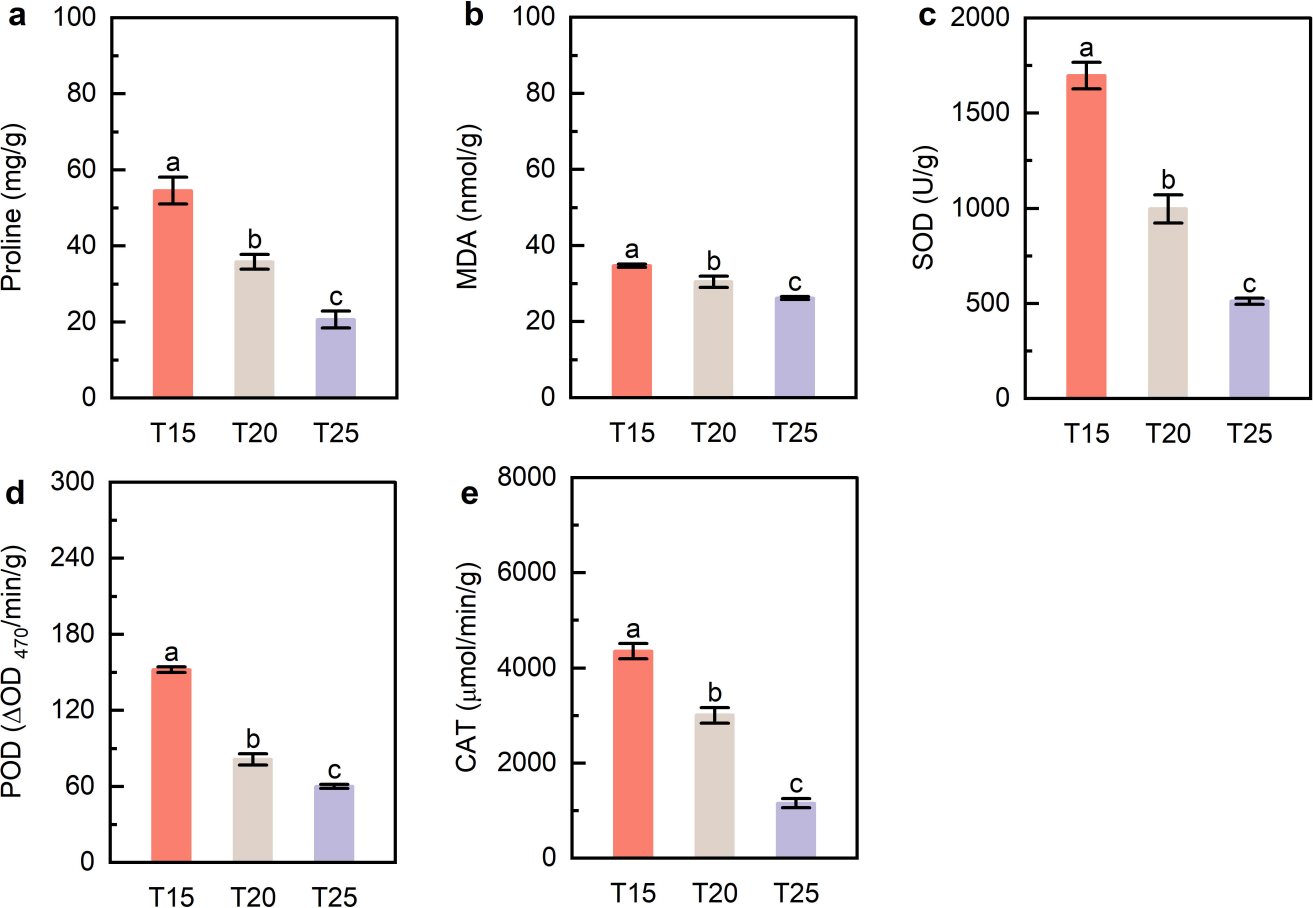
Leaf proline content, malondialdehyde content, and antioxidant enzyme activities. **(a)** Proline content; **(b)** MDA, malondialdehyde; **(c)** SOD, superoxide dismutase; **(d)** POD, peroxidase; **(e)** CAT, catalase. T15, T20, and T25 represent plants grown under different day/night temperature regimes of 15/5°C, 20/10°C, and 25/15°C, respectively. Different lowercase letters indicate significant differences among treatments (*p* < 0.05).

Leaf proline and MDA contents decreased with increasing temperature, with T25 showing significantly lower levels than T20 and T15 (**Figures 3a, b**). Similarly, SOD, POD, and CAT activities were markedly reduced at T25 compared with the lower-temperature treatments (**Figures 3c–e**). These results indicate that moderate warming reduces both osmolyte accumulation and antioxidant enzyme activity.

### 3.4 Concentrations of active compounds in leaves

**Figure 4:**
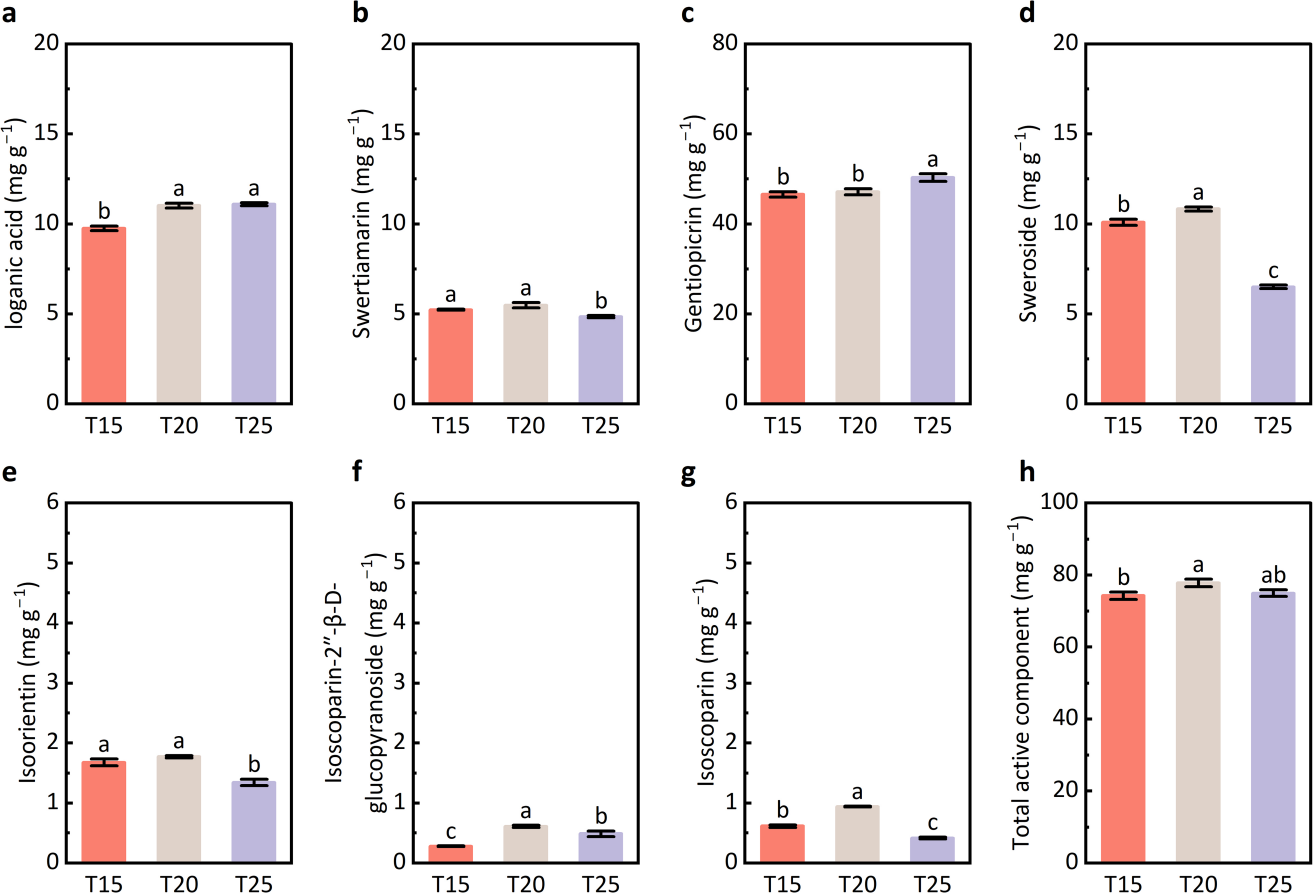
Concentrations of bioactive compounds in leaves of *G. farreri*. **(a)** Loganic acid; **(b)** Swertiamarin; **(c)** Gentiopicrin; **(d)** Sweroside; **(e)** Isoorientin; **(f)** Isoscoparin-2″-β-D-glucopyranoside; **(g)** Isoscoparin; **(h)** Total active compound concentration. T15, T20, and T25 represent plants grown under different day/night temperature regimes of 15/5 °C, 20/10 °C, and 25/15 °C, respectively. Different lowercase letters indicate significant differences among treatments (*p* < 0.05).

The contents of loganic acid and gentiopicrin in leaves were significantly higher under T25 than under T15 (**Figures 4a,c**). In contrast, the concentrations of swertiamarin, sweroside, isoorientin, and isoscoparin were significantly lower under T25 compared with T15 and T20 (**Figures 4b, d, e, g**). No significant differences in total bioactive compound content were observed among the temperature treatments (**Figure 4h**). Overall, increasing temperature (T25) promoted the accumulation of two iridoid glycosides (loganic acid and gentiopicrin), while reducing the levels of two iridoid glycosides (swertiamarin and sweroside) and two flavonoids (isoorientin and isoscoparin), without affecting the total bioactive compound content.

### 3.4 Transcriptome analysis

**Figure 5:**
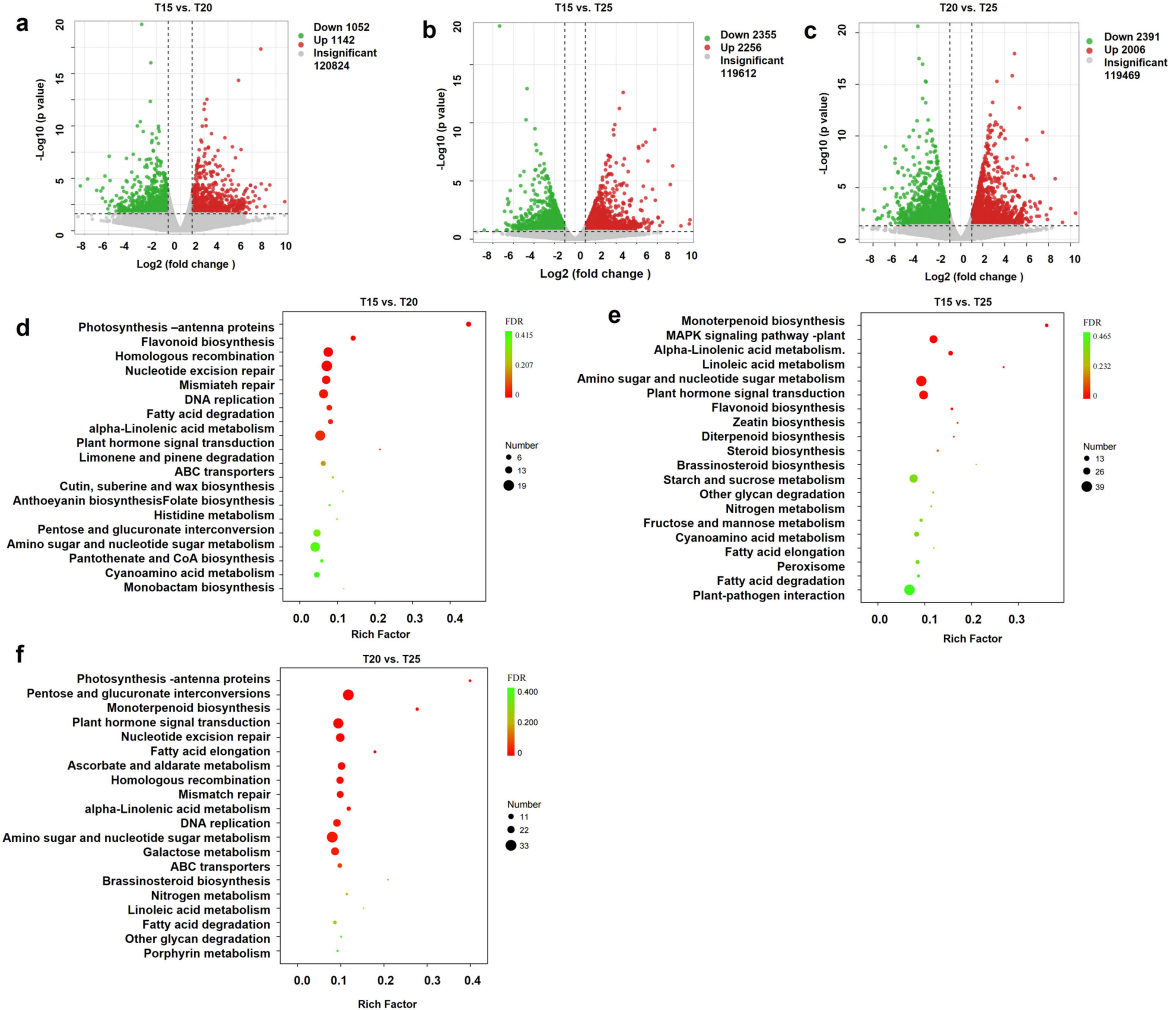
Differentially expressed genes and KEGG pathway enrichment analysis. **(a, d)** Volcano plot of DEGs and KEGG pathway enrichment for T15 vs. T20. **(b, e)** Volcano plot of DEGs and KEGG pathway enrichment for T15 vs. T25. **(c, f)** Volcano plot of DEGs and KEGG pathway enrichment for T20 vs. T25. T15, T20, and T25 represent plants grown under different day/night temperature regimes of 15/5 °C, 20/10 °C, and 25/15 °C, respectively.

To elucidate gene expression changes in *G. farreri* under warming conditions, transcriptome analysis was performed. DEGs varied across temperature treatments (**Figures 5a–c**). Compared with T15, 2,194 genes were differentially expressed in T20 (1,142 upregulated, 1,052 downregulated), and 4,611 in T25 (2,256 upregulated, 2,355 downregulated). Between T20 and T25, 4,397 DEGs were identified (2,006 upregulated, 2,391 downregulated).

KEGG enrichment analysis of DEGs highlighted significant pathways, with the top 20 pathways for each comparison visualized. DEGs were highly enriched in Photosynthesis-antenna proteins, Flavonoid biosynthesis, Monoterpenoid biosynthesis, Plant hormone signal transduction, and Amino sugar and nucleotide sugar metabolism (**Figures 5d–f**).

### 3.5 Weighted gene co-expression network analysis

**Figure 6:**
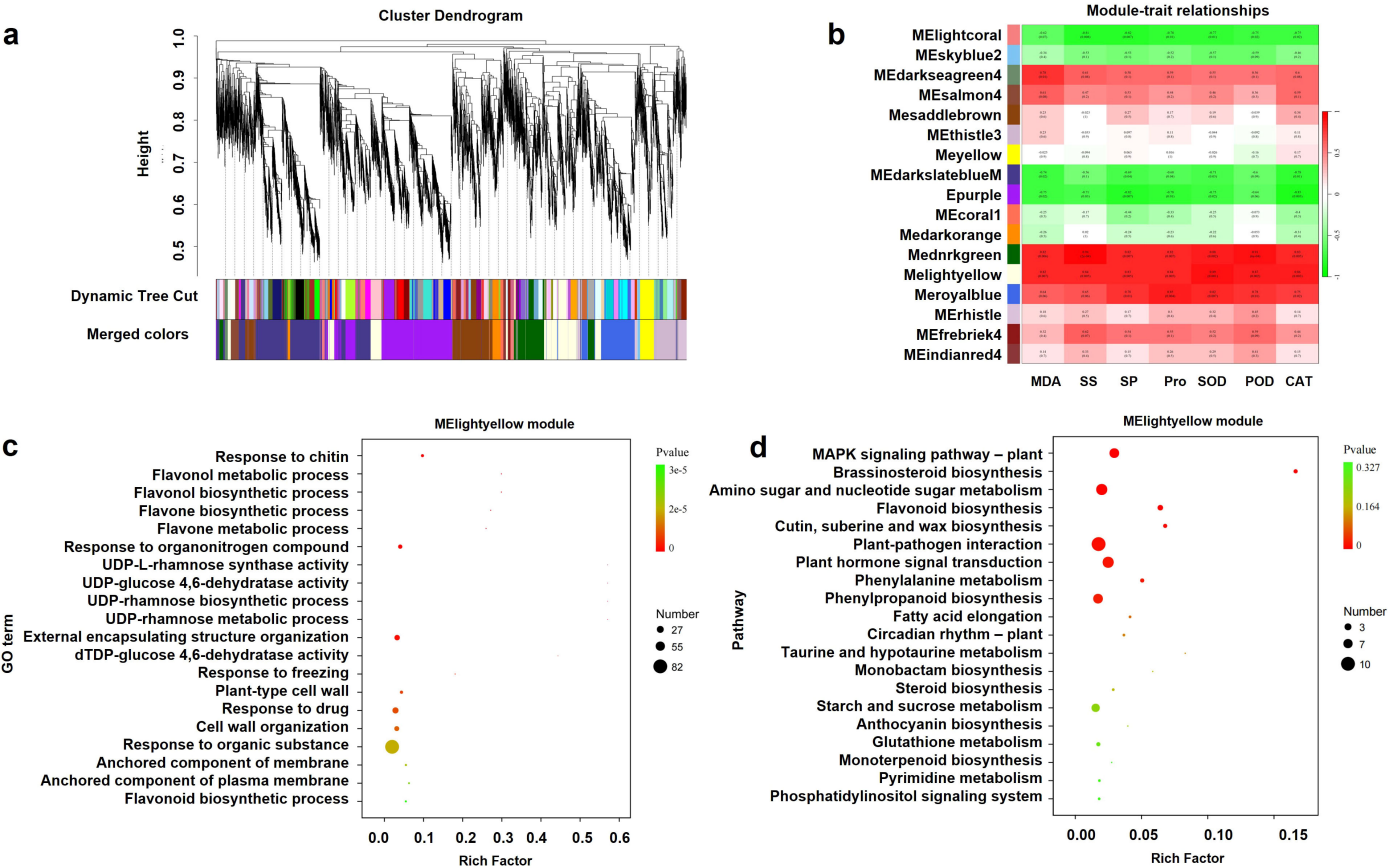
Weighted gene co-expression network analysis. **(a)** WGCNA module classification. **(b)** Heatmap showing correlations between modules and physiological traits. **(c)** Gene Ontology (GO) enrichment analysis of the MElightyellow module. **(d)** KEGG pathway enrichment analysis of the MElightyellow module. T15, T20, and T25 represent plants grown under different day/night temperature regimes of 15/5 °C, 20/10 °C, and 25/15 °C, respectively.

To investigate temperature-responsive regulatory networks in *G. farreri* seedlings, DEGs were integrated with seven key stress-related physiological traits (MDA, soluble sugar, soluble protein, proline, SOD, POD, CAT) for WGCNA. The Darkslateblue module contained the most DEGs (1,626), followed by Purple (1,527), while FIREBRICK4 contained the fewest (40) (**Figure 6a**). Module–trait correlation analysis revealed strong associations: MDA with MEdarkgreen and MElightyellow (r = 0.82), soluble sugar with MEdarkgreen (r = 0.94), soluble protein with MElightyellow (r = 0.83), proline with MEroyalblue (r = 0.85), SOD with MElightyellow (r = 0.89), POD with MEdarkgreen (r = 0.91), and CAT with MElightyellow (r = 0.86) (**Figure 6b**). MElightyellow, significantly correlated with multiple traits, was identified as a key module under low-temperature stress.

GO enrichment of MElightyellow DEGs highlighted processes including response to chitin, flavonol metabolism, and flavonol biosynthesis (**Figure 6c**). KEGG analysis revealed significant enrichment in MAPK signaling, brassinosteroid biosynthesis, amino sugar and nucleotide sugar metabolism, flavonoid biosynthesis, cutin/suberin/wax biosynthesis, and plant–pathogen interaction, with the latter showing the highest number of DEGs (10) (**Figure 6d**). Consequently, the plant–pathogen interaction pathway was selected for further analysis.

### 3.6 Differential gene expression in the plant–pathogen interaction pathway

**Figure 7:**
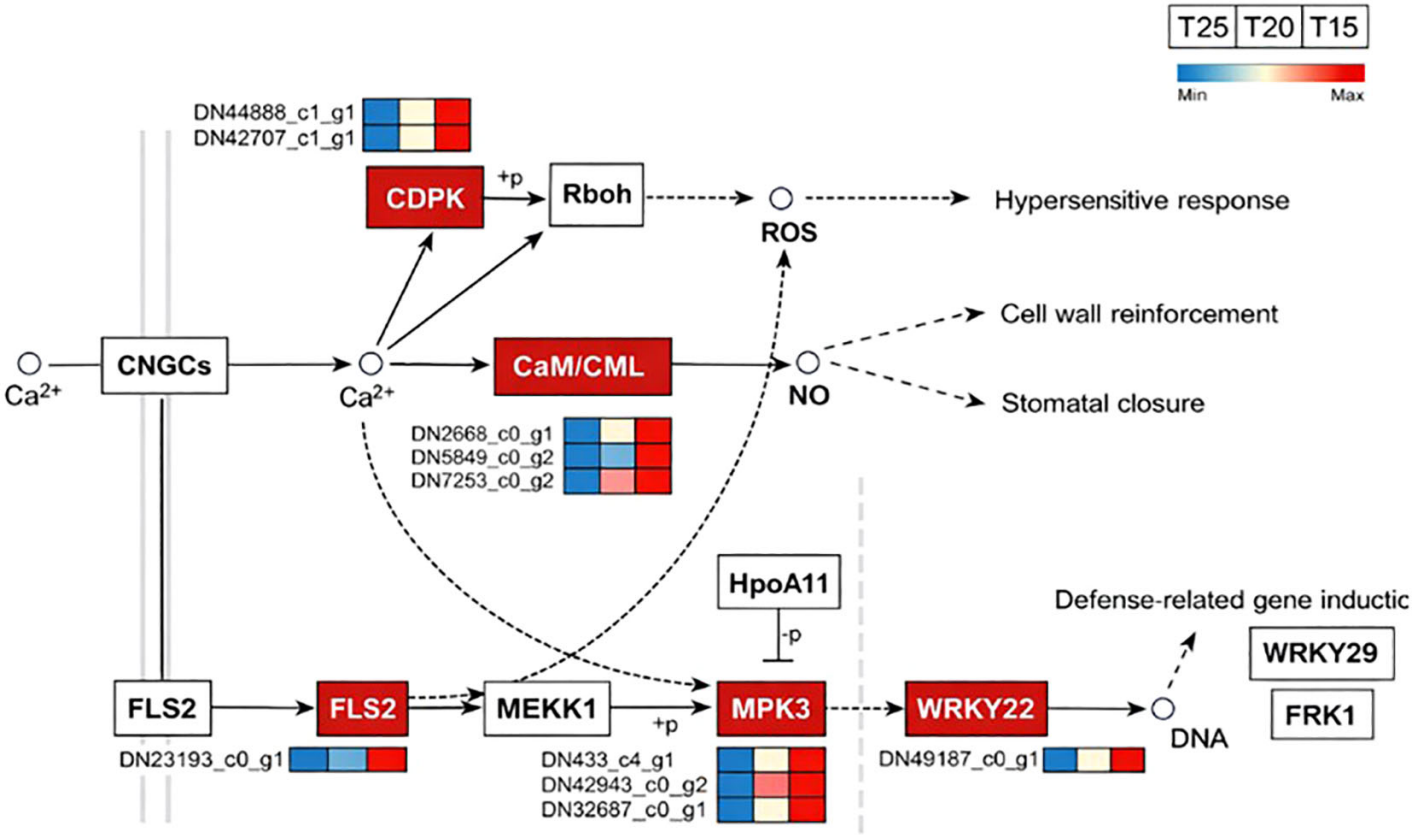
Differentially expressed genes involved in the plant–pathogen interaction pathway. T15, T20, and T25 represent plants grown under different day/night temperature regimes of 15/5 °C, 20/10 °C, and 25/15 °C, respectively.

Analysis of the plant–pathogen interaction pathway identified five key regulatory nodes: FLS2, CDPK, CaM/CML, MPK3, and WRKY22, all of which were upregulated under T15. Within calcium signaling, one FLS2 gene (DN23193_c0_g1) and two CDPK genes (DN44888_c1_g1, DN42707_c1_g1) were significantly differentially expressed, potentially triggering ROS production to activate hypersensitive responses and stress tolerance. Expression of these genes was highest at T15 and lowest at T25. Three CaM/CML genes (DN2668_c0_g1, DN7253_c0_g2, DN5849_c0_g1) were also significantly upregulated at T15, likely promoting NO synthesis, reinforcing cell walls, and inducing stomatal closure. Similarly, MPK3 (DN433_c4_g1, DN42943_c0_g2, DN32687_c0_g1) and WRKY22 (DN49187_c0_g1) showed consistent expression patterns, peaking at T15 and declining at T25 (**Figure 7**).

### 3.7 Metabolomic analysis

**Figure 8:**
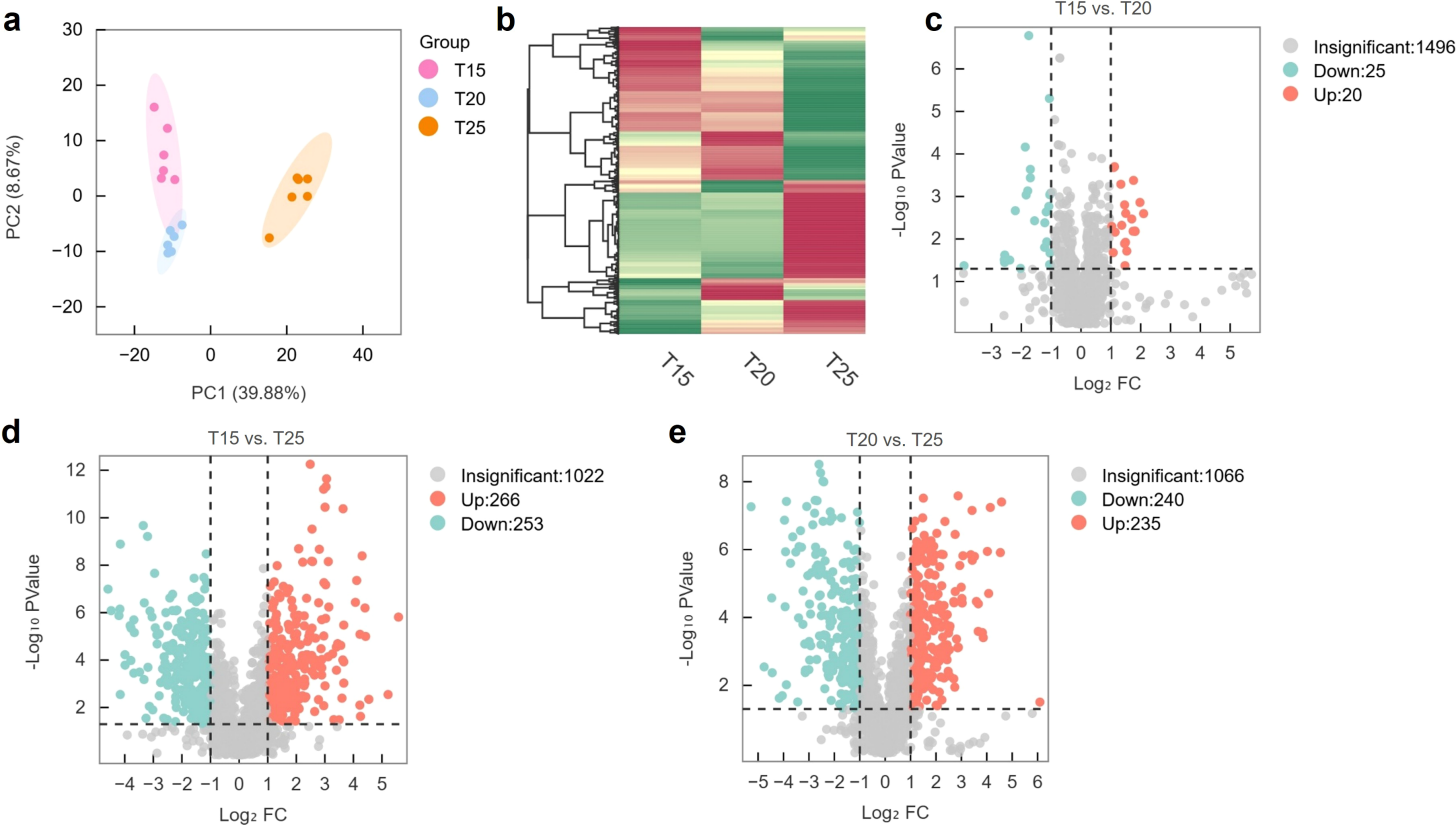
Metabolomic analysis. **(a)** Principal component analysis score plot of samples. **(b)** Hierarchical clustering of metabolites. **(c)** Differential metabolites between T15 and T20. **(d)** Differential metabolites between T15 and T25. **(e)** Differential metabolites between T20 and T25. T15, T20, and T25 represent plants grown under different day/night temperature regimes of 15/5 °C, 20/10 °C, and 25/15 °C, respectively.

Principal component analysis (PCA) showed clear separation of T25 from T20 and T15, while T15 and T20 clustered closely, indicating greater metabolic divergence at moderate warming (**Figure 8a**). Across treatments, 1,541 metabolites were identified and grouped into four clusters based on accumulation patterns. Compared with T15, 25 metabolites were upregulated and 20 downregulated in T20; 266 were upregulated and 253 downregulated in T25. Between T20 and T25, 240 metabolites were upregulated and 235 downregulated (**Figures 8c–e**), demonstrating extensive temperature-dependent metabolic reprogramming.

### 3.8 Flavonoid biosynthesis: SDMs and DEGs

**Figure 9:**
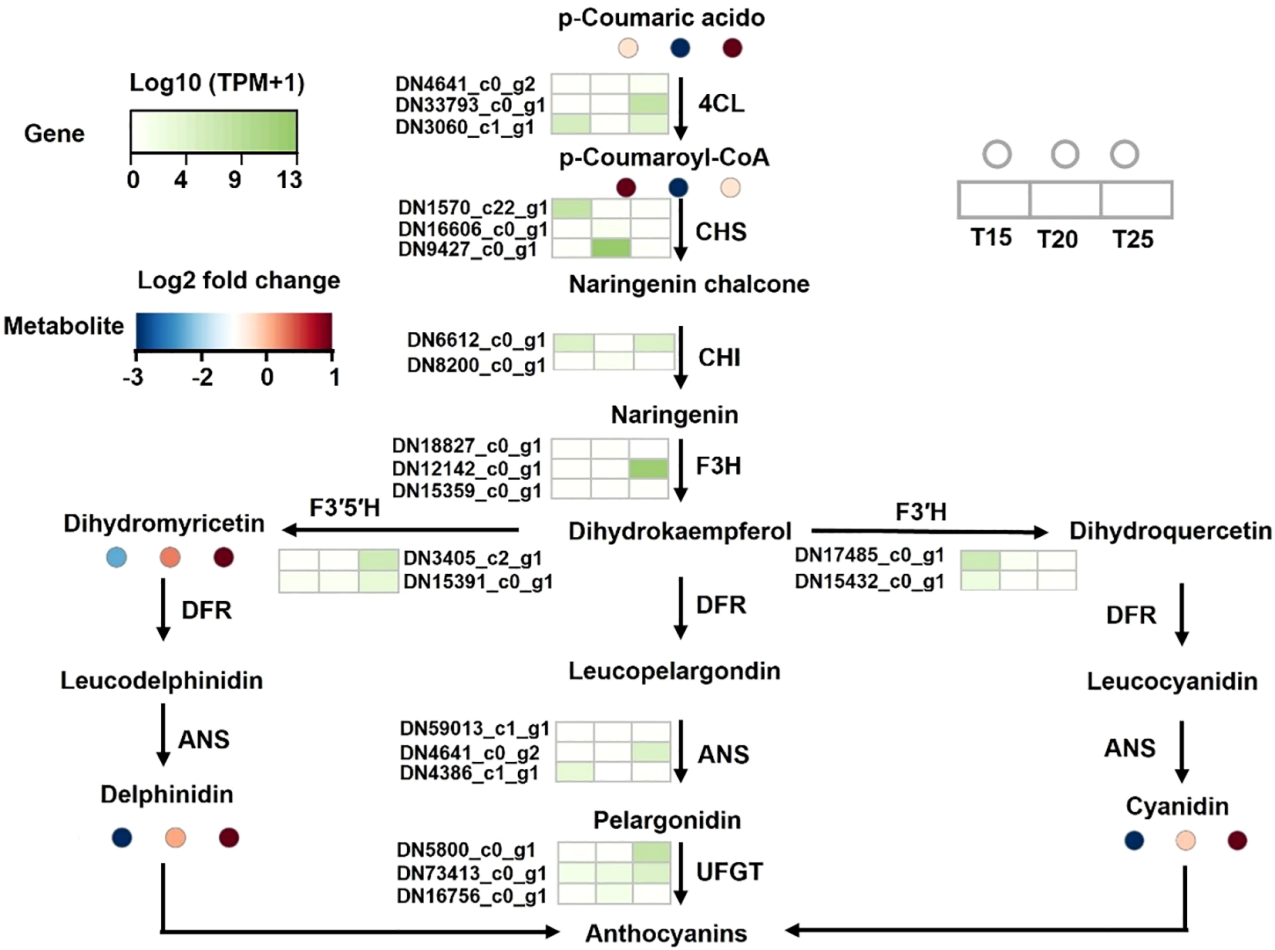
SDMs and DEGs involved in flavonoid biosynthesis. 4CL, 4-coumarate:CoA ligase; CHS, chalcone synthase; CHI, chalcone isomerase; F3H, flavanone 3-hydroxylase; F3′H, flavonoid 3′-hydroxylase; DFR, dihydroflavonol 4-reductase; ANS, anthocyanidin synthase; UFGT, UDP-glucose:flavonoid 3-O-glucosyltransferase. T15, T20, and T25 represent plants grown under different day/night temperature regimes of 15/5 °C, 20/10 °C, and 25/15 °C, respectively.

Temperature influenced both flavonoid accumulation and the expression of key biosynthetic genes. At T25, levels of *p*-coumaric acid, dihydromyricetin, delphinidin, and cyanidin were higher than at T20 or T15. Correspondingly, genes encoding 4CL (DN33793_c0_g1), F3H (DN12142_c0_g1), F3′5′H (DN3405_c2_g1), ANS (DN4641_c0_g2), and UFGT (DN5800_c0_g1) were upregulated at T25 relative to the lower-temperature treatments (**Figure 9**), indicating coordinated transcriptional regulation of flavonoid biosynthesis under moderate warming.

## 4 Discussion

### 4.1 Increased temperature promotes biomass accumulation and growth in *G. farreri*

Plant growth responses to temperature are strongly shaped by species-specific thermal adaptation and environmental origin (Fu et al., 2015). Alpine plants are typically constrained by low temperature in their native habitats, and moderate warming can relax these limitations, thereby enhancing growth performance (Shi et al., 2010). Meta-analyses and field warming experiments have shown that temperature elevation often promotes biomass accumulation in cold-limited ecosystems, although responses vary among species and functional groups (Fu et al., 2013).

In the present study, moderate warming under low-altitude conditions significantly promoted vegetative growth in *G. farreri*. Increases in leaf number, leaf area, shoot biomass, and root biomass were consistently observed at 25/15 °C compared with cooler temperature regimes (**Figures 1b, c, h, i**). These coordinated morphological responses indicate that moderate warming enhances both aboveground and belowground growth, supporting improved whole-plant carbon acquisition and allocation.

Leaf chlorophyll content increased under warmer conditions, whereas soluble sugar and soluble protein concentrations declined (**Figures 2c, e, f**). Rather than indicating reduced carbon assimilation, this pattern likely reflects enhanced growth-driven utilization and redistribution of photosynthates. Larger leaf area and higher chlorophyll content are generally associated with increased photosynthetic capacity at the canopy level, even when carbohydrate concentrations per unit tissue decrease due to accelerated growth and metabolic demand. Similar patterns have been reported in other cold-adapted species, where warming stimulates leaf expansion and biomass accumulation while reducing the accumulation of soluble carbohydrates in leaves (Klein et al., 2008; Bjorkman et al., 2018; Fazlioglu and Wan, 2021). However, opposite trends have also been documented. In alpine species such as *Pedicularis punctata* and *Plantago major*, increasing temperature along decreasing altitude gradients led to elevated leaf sugar contents (Rahman et al., 2020), suggesting that carbohydrate responses to warming are species-dependent and may reflect differences in source–sink dynamics or metabolic regulation. In *G. farreri*, the simultaneous increase in biomass and decrease in soluble metabolites supports the interpretation that moderate warming enhances carbon use efficiency rather than limiting photosynthetic performance.

Root growth was also markedly enhanced under warmer conditions, as evidenced by increased root length and root dry weight. Enhanced root development may improve nutrient and water acquisition, further reinforcing shoot growth and overall plant performance under low-altitude cultivation. Collectively, these results indicate that moderate warming alleviates temperature limitation on growth in *G. farreri*, promoting coordinated increases in photosynthetic structure, biomass accumulation, and carbon allocation. This growth-oriented response provides a physiological basis for the successful *ex situ* cultivation of alpine medicinal plants under moderately moderate warming.

### 4.2 Effects of temperature on antioxidant capacity

Temperature modulates plant antioxidant capacity through coordinated regulation of enzyme activities, gene expression, and genotype-dependent responses. Antioxidant enzymes constitute a primary defense against oxidative stress by scavenging excess reactive oxygen species (ROS) generated under temperature stress (Naudts et al., 2014). Under high-temperature stress, enhanced activities of SOD, POD, CAT, and APX have been widely reported and are closely associated with improved thermotolerance in multiple species, including lettuce, white clover, and transgenic tobacco (Li et al., 2020; Liu et al., 2021; Singh et al., 2021). Conversely, increased antioxidant enzyme activity and reduced MDA accumulation are also characteristic of cold-tolerant genotypes under low-temperature stress (Ramazan et al., 2021).

In high altitudinal plants, temperature-driven changes in antioxidant responses are often species- and context-dependent. Warming enhanced antioxidant enzyme activities while increasing MDA accumulation in *Leymus secalinus*, indicating intensified membrane lipid peroxidation (Shen et al., 2019), whereas in *Elymus nutans*, warming reduced MDA content but increased SOD and APX activities (Shi et al., 2010). Such contrasting responses highlight that antioxidant regulation depends on stress intensity, duration, and inherent stress tolerance.

In the present study, increasing temperature to 25 °C significantly reduced SOD, POD, and CAT activities, accompanied by decreased MDA accumulation in *G. farreri* leaves (**Figures 3b–e**). These results suggest that 25 °C alleviated oxidative stress relative to 15 °C, thereby lowering the demand for antioxidant defence. Collectively, our findings indicate that moderate warming does not impose heat stress on *G. farreri*, but instead reflects its strong temperature tolerance and reduced ROS pressure under warmer growth conditions.

### 4.3 Effects of temperature on the plant–pathogen interaction associated pathway

The plant–pathogen interaction pathway integrates a range of signaling components that are broadly involved in plant responses to environmental cues, including perception mechanisms and downstream signal transduction processes (Dodds and Rathjen, 2010; Wirthmueller et al., 2013). Increasing evidence suggests that abiotic factors such as temperature variation can substantially influence this pathway by reprogramming hormone signaling, redox balance, and stress-responsive gene expression, thereby shaping overall plant performance under changing environments (Pandey and Senthil-Kumar, 2019). Temperature effects on this pathway have been reported across plant species, with moderate warming (25–35 °C) altering signaling dynamics and physiological outcomes (Uauy et al., 2005; Webb et al., 2010; Kumar et al., 2024).

In this study, WGCNA revealed that multiple stress-related physiological traits were significantly associated with the MElightyellow module (**Figure 6b**). KEGG enrichment analysis showed that genes annotated to the plant–pathogen interaction pathway were overrepresented within this module (**Figure 6d**). Notably, several key signaling components, including FLS2 and CDPK genes, displayed temperature-dependent expression patterns, with higher transcript abundance under low-temperature conditions and reduced expression under moderate warming (T25) (**Figure 7**).

Given that FLS2 and CDPKs are known to participate in Ca^2+^-mediated signal transduction and redox-related signaling processes, their coordinated downregulation under warmer conditions likely reflects an alleviation of stress-associated signaling rather than activation of defense responses. This interpretation is supported by the concurrent decline in antioxidant enzyme activities and lipid peroxidation levels observed at higher temperatures, suggesting a reduced requirement for stress-induced signaling and protective responses.

These results indicate that moderate warming modulates stress-associated signaling pathways involving FLS2–CDPK regulatory nodes, thereby reshaping the physiological state of *G. farreri*. Rather than triggering defense-related responses, moderate warming appears to relieve low-temperature constraints, leading to a downregulation of stress-responsive signaling and facilitating growth and secondary metabolite accumulation. This temperature-dependent adjustment of signaling networks may represent an important mechanism underlying the acclimation of altitudinal medicinal plants to low-altitude cultivation environments.

### 4.4 Warming enhances flavonoid metabolism in *G. farreri*

Flavonoid biosynthesis is tightly regulated by environmental cues, with temperature emerging as a major determinant of pathway activity. Under thermal stress, plants frequently enhance the production of antioxidant secondary metabolites, including flavonoids and phenolic compounds, to mitigate oxidative damage (Ghasemzadeh et al., 2010; Cheng et al., 2018). Altitudinal species are particularly enriched in flavonoids, which contribute substantially to redox homeostasis and stress tolerance (Vitalini et al., 2016; Li et al., 2024). In the medicinal plant *Justicia adhatoda* L., individuals growing at higher altitudes exhibit increased contents of phenolic and flavonoid compounds (Jamwal et al., 2023). Temperature-dependent modulation of flavonoid metabolism has been widely reported, often involving activation of the phenylpropanoid pathway and increased expression of key biosynthetic enzymes such as PAL and 4CL (Janas et al., 2002; Lillo et al., 2008; Yang et al., 2018; Guo et al., 2020). However, how moderate warming affects flavonoid metabolism in cold-adapted medicinal plants during low-altitude cultivation remains poorly understood.

In the present study, metabolomic analysis revealed that moderate warming (25/15 °C) significantly increased the accumulation of several flavonoid compounds, including *p*-coumaric acid, dihydromyricetin, delphinidin, and cyanidin, compared with cooler temperature regimes. Consistently, transcriptomic data showed elevated expression of key flavonoid biosynthetic genes, including 4CL, F3H, F3′5′H, ANS, and UFGT, under warmer conditions (**Figure 9**). These coordinated changes at both the metabolite and transcript levels indicate that moderate warming promotes flavonoid biosynthesis through enhanced pathway activity rather than isolated metabolic shifts.

Notably, the increase in flavonoid accumulation coincided with reduced antioxidant enzyme activities and lower levels of lipid peroxidation, suggesting that flavonoid production under warmer conditions may reflect a metabolic reallocation associated with alleviated stress rather than a compensatory response to oxidative damage. This pattern supports the view that flavonoids in *G. farreri* contribute to metabolic acclimation during the transition from cold native habitats to warmer low-altitude environments. These findings demonstrate that moderate warming reshapes secondary metabolism in *G. farreri* by enhancing flavonoid biosynthesis, likely through temperature-sensitive regulation of the phenylpropanoid pathway. Such metabolic plasticity may facilitate the successful *ex situ* cultivation of alpine medicinal plants under climate warming scenarios by simultaneously supporting growth.

In *G. farreri*, the major active compounds include flavonoids and iridoid glycosides. Notably, moderate warming did not significantly affect the total content of these bioactive compounds (**Figure 4h**). However, the accumulation of specific flavonoids, including isoorientin and isoscoparin, was significantly reduced under T25 (**Figures 4e,g**). Metabolomic analysis further revealed that differentially accumulated metabolites were significantly enriched in the flavonoid biosynthesis pathway (**Supplementary Figures S1a, b**). These results suggest that moderate warming alters flavonoid metabolic flux, whereby a greater proportion of flavonoids may be allocated to growth-related processes, while the accumulation of active flavonoids is reduced.

## 5 Conclusions

**Figure 10:**
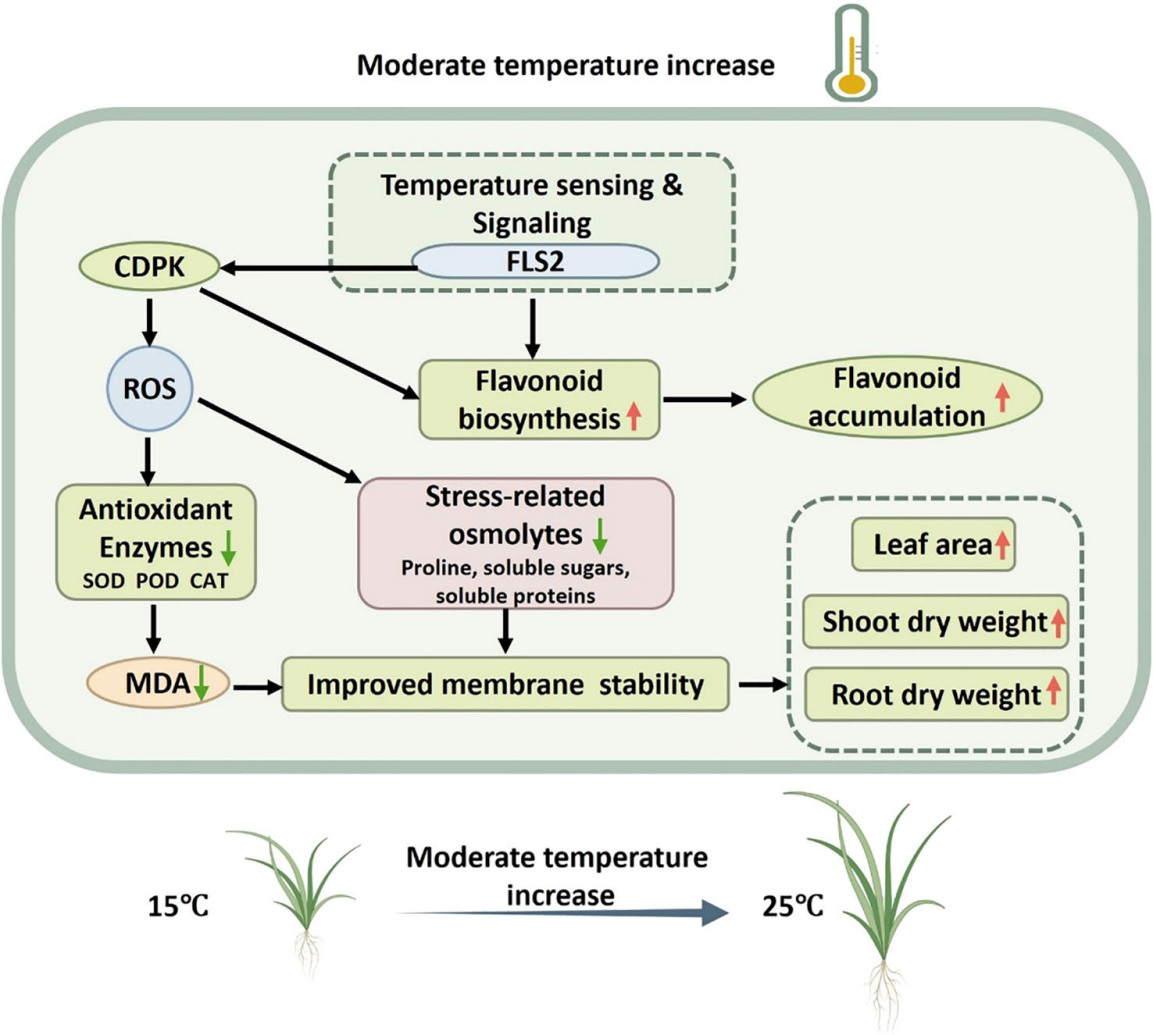
Schematic model illustrating the regulation of antioxidant systems and flavonoid biosynthesis in *G. farreri* in response to temperature changes.

Understanding plant responses to environmental change is fundamental for the *ex situ* cultivation of altitudinal medicinal species. *G. farreri*, a plant native to cold, high-altitude habitats, exhibited improved growth performance under moderately moderate warming at low altitude. Warming significantly increased leaf number, leaf area, root biomass, and chlorophyll content. Concurrently, antioxidant enzyme activities declined, while the plant–pathogen interaction pathway emerged as a major stress-related regulatory pathway, with FLS2–CDPK identified as key regulatory nodes. In addition, flavonoid accumulation was markedly enhanced under warmer conditions (**Figure 10**). Collectively, these results demonstrate that moderate warming alleviates stress constraints and promotes both growth and secondary metabolite accumulation in *G. farreri*. Our findings provide insights into temperature-driven physiological and metabolic acclimation in altitudinal medicinal plants and offer a scientific basis for optimizing low-altitude *ex situ* cultivation strategies under ongoing climate warming. However, the detailed regulatory mechanisms underlying temperature-dependent flavonoid biosynthesis, including enzymatic activity, transcriptional control, and associated signaling networks, warrant further investigation.

## Data Availability

The Illumina sequencing data generated in this study have been made publicly available. The data can be accessed at: https://www.ncbi.nlm.nih.gov/sra/PRJNA1456657.
