# Supplementary material for: Moderate warming promotes growth and flavonoid biosynthesis in the altitudinal medicinal plant Gentiana lawrencei var. farreri
Source: Front Plant Sci. 2026 Apr 24;17:1806606. doi: 10.3389/fpls.2026.1806606 (PMC13154219; doi:10.3389/fpls.2026.1806606)
Supplement: Supplementary file 1 [file SupplementaryFile1.docx]

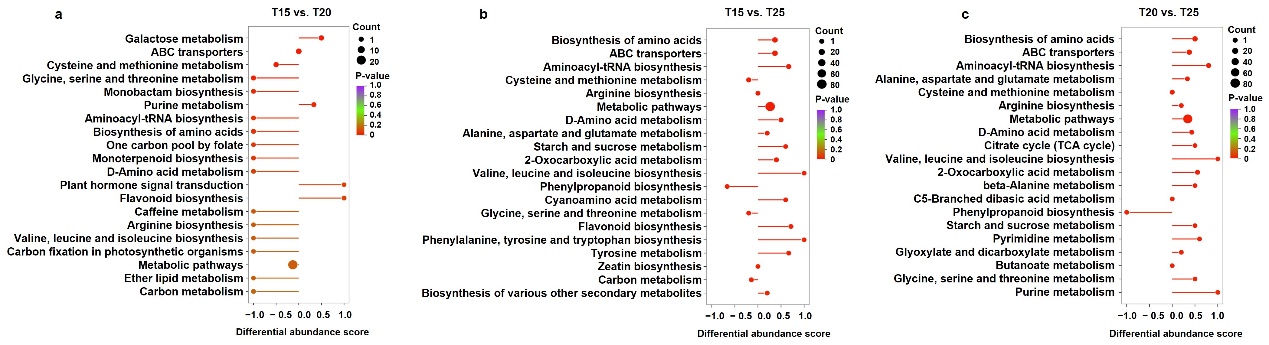


Fig. S1 KEGG pathway enrichment analysis of differentially accumulated metabolites. (a) Enrichment analysis of metabolites in T15 vs. T20; (b) T15 vs. T25; (c) T20 vs. T25. T15, T20, and T25 represent plants grown under day/night temperature regimes of 15/5 °C, 20/10 °C, and 25/15 °C, respectively.
